# Sampling immigrants in the Netherlands and Germany

**DOI:** 10.1186/s40878-017-0062-2

**Published:** 2017-12-15

**Authors:** Kurt Salentin, Hans Schmeets

**Affiliations:** 1 Bielefeld University, Institute for Interdisciplinary Research on Conflict and Violence, Universitätsstraße 25, 33615 Bielefeld, Germany; 2 Maastricht University / Statistics Netherlands, Faculty of Arts and Social Sciences, Grote Gracht 90-92, 6211 SZ Maastricht, The Netherlands

**Keywords:** Sampling, Comparative research, Immigrants, The Netherlands, Germany, Population register, Harmonisation

## Abstract

This paper discusses the limitations of harmonised sampling designs for survey research on immigrants in Germany and the Netherlands. Although the concepts for immigrants are largely similar in both countries, there are severe constraints when it comes to comparable sampling designs. While in the Netherlands a sample can be drawn from a national population register by Statistics Netherlands, this is impossible in Germany due to the decentralised setup of the population register and legal restrictions on merging existing databases. Harmonisation of immigrant statistics is thus less a problem at the concept level than in the implementation. Achieving a harmonised data collection on immigrants for Germany and the Netherlands will be a major challenge.

## Introduction

The aim of this article is to provide researchers interested in comparative migration and minority studies an introduction to the potential and the limitations of sampling migrant and minority populations in the Netherlands and Germany. Harmonized data-collection is crucial for a comparison of survey results across countries (Huddleston, Niessen, & Tjaden, 2013). Two major challenges will be discussed: (1) to understand the concepts used and the groups to which they are applied; and (2) to implement representative samples of the target populations. Migration and integration researchers in the academic field and in official statistics are the intended audience. The two countries are selected because they are not only geographical neighbours but also comparable in several ways. Both are export-oriented industrial economies with a history of intense labour recruitment after World War II and they have migrants from important countries of origin in common. Their concepts of immigrants are comparable and in both countries one in five people is part of an immigrated family. Both have comprehensive population registers that can serve as sampling frames.

However, in many fields, including immigrants, the statistical figures are not harmonised between Germany and the Netherlands. One important reason is the lack of identical sampling frames. In this paper we will address the challenges of drawing samples based on immigrants living in the Netherlands and Germany. Before the sampling strategies will be discussed, we will outline the various concepts referring to immigrants, and their implementation. It will be demonstrated that in both countries traditional randomized sampling techniques among immigrants necessitate an adjustment of the population definition as many immigrants are not (yet) registered and consequently are not found in sampling frames. Furthermore, the two countries differ in the possibilities to use population registers for sampling designs and link registers enabling harmonised sampling strategies. We will start with a short overview of the immigrants, followed by the concepts and sampling techniques used. In the conclusions, we will provide an answer to which extent harmonised sampling strategies are feasible and the consequences for survey research.

## Immigrants in The Netherlands and Germany

In line with many other European countries, the Netherlands has a relatively short history of immigration. Large-scale immigration occurred in earlier centuries, but very little between 1850 and 1950. From 1950s onwards, high demand triggered a wave of labour immigration. The perception was basically that the immigrants, called guest laborers, would return to their country of origin. Most immigrants came from southern Europe, mainly Spain and Turkey, together with immigrants from Morocco. After this influx of immigrants, many (Dutch) people from Surinam and the Netherlands Antilles came, followed by the refugees from Eastern European countries (e.g. Bosnia), Iraq, Somalia, and Afghanistan. More recently, immigrants from EU-countries Poland, Bulgaria, Romania and from Syria entered the Netherlands. Immigrants who stay longer than 4 months are required to register in the municipality they moved to. From 1950 onwards, the yearly registered immigrant numbers in the Municipal Personal Records Database (BRP) have almost tripled: from 71 thousand in 1950 to 205 thousand in 2015. These include also Dutch citizens who returned to the Netherlands after a stay abroad. Note that in the 1950–2015 time span, the total population almost doubled to 17.0 million, including 3.8 million immigrants (first and second generation). Most originate from Turkey, Morocco, Indonesia, Germany and Surinam (350 to 400 thousand per country of origin). Also a substantial number (over 100 thousand) originate from Aruba and the former Netherlands Antilleans, Poland, and Belgium. Detailed information as to age/gender, regional distribution, urbanity, residence duration are available, all based on register information (see: www.cbs.nl; various Statline tables).^1^

Germany has always been a country of immigration and emigration, with poverty, persecution, wars, industrialisation and economic growth as push and pull factors (Bade, Emmer, Lucassen, & Oltmer, 2010). After World War II, population movements reached unprecedented numbers. 12.5 million displaced persons from Eastern Europe arrived in the two German states in the direct aftermath of the war and large numbers of labourers abducted by the Nazi regime remained. From 1955 on, 14 million industrial labourers and their families from Mediterranean countries were recruited to the Federal Republic of Germany and from Vietnam and Mozambique to the German Democratic Republic. Despite high remigration rates, 5.3 million persons from Turkey, Italy and former Yugoslavia and other countries became permanent residents (Statistisches Bundesamt, 2016, p. 62). In the 1980s, 20,000 to 120,000 people from Vietnam, Sri Lanka, Iran and other war-torn states sought asylum in Germany each year. In the 1990–2016 period, a total of 4.4 million people from regions such as former Yugoslawia applied for asylum (Bundesamt für Migration und Flüchtlinge, 2017, p. 3). In 2016 alone, 890,000 refugee arrivals were registered,^2^ mainly by Syrians, Iraqis, Afghans and other persons from Africa and the Arabian peninsula. 4.5 million Aussiedler of German descent from the Soviet Union, Poland and Romania mainly arrived between 1989 and 1999 (Bundesverwaltungsamt, 2016, p. 5). Meanwhile the eastward expansion of the European Union attracted migrants from Poland, Romania and Bulgaria, along with a general increase of migration in conjunction with intra-European freedom of mobility. New migrant categories comprise foreign spouses to Germans from Eastern Europe, Southeast Asia, and Latin America (Aybek, Babka von Gostomski, & Rühl, 2013).

In short, both countries show many similarities in the influx of immigrants and the total share of immigrants. However, history reveals that the countries of origin differ, indicating that for a comparison between the Netherlands and Germany, the composition of the immigrants is an important aspect to deal with. In the next section the concepts used in both countries will be compared.

## Concepts

The collection of comparable, harmonised, international data becomes more important due to the salience of migration at the political level after 1989 (Kraler, Reichel, & Entzinger, 2015, p. 41). In 1989 the International Organisation for Migration (IOM), the successor of the Intergovernmental Committee for Migration (ICM), was established as expert organisation, and in 1990 the office of the United Nations High Commissioner for Refugees (UNHCR) was expanded. In addition, from the perspective of the 2030 Sustainability Development Goals, the United Nations Office of the High Commissioner for Human Rights (OHCHR) recommends to collect and publish official statistical data disaggregated by ethnicity and migration (OHCHR, 2016, p. 6). Such data should be based on self-identification, rather than through imputation or proxy (OHCHR, 2016, p. 8).

Harmonising international migration statistics are guided by regulations from the European Commission, such as 862/2007, adopted in July 2007, which is one of the cornerstones of migration statistics policy in the EU. Eurostat, the EU statistical office, is in charge to implement such regulations in close collaboration with the National Statistical Offices, including Statistics Netherlands and the Statistisches Bundesamt. According to regulation 862/2007, data collection was no longer voluntary on issues of migration and asylum, and furthermore definitions were provided as to various concepts, such as citizenship, country of birth, asylum, and residence permits. However, despite of efforts in providing guidelines, countries often differ not only in the data-collection and weighting model to reduce the non-response bias, but also in the operationalization of concepts such as migrants or minorities, and in the phrasing of the questions in their surveys, including the EU-Labor Force Survey (EU-LFS) and the EU-Survey on Income and Living Conditions (Huddleston et al., 2013, p. 33). Such concepts to define migrants may also differentiate along the perspective of interest: nationality (citizenship), origin, residence or migration history, legal status, descent and ethnicity (Kraler et al., 2015, pp. 50–51).

In international statistics an ‘immigrant’ is a catch-all category which can refer to ‘country of birth’ , ‘citizenship’ or ‘country of birth of one or both parent(s)’ (Huddleston et al., 2013, p. 39). In the EU-LFS an ad hoc module on migrants in the labor market was implemented in 2008, and repeated in 2014. Data on the ‘foreign background’ or ‘migrant background’ were collected in a large number of EU member states, often referring to residents of which both parents were born abroad, capturing first and second generation. In addition, data on citizenship (or ‘nationality’ as it is often termed in international law) were collected, which refers to a *legal status* that indicates the relation between an individual and a state (Bauböck, 1994, p. 23; Kivisto & Faist, 2007, pp. 1–2). Hence, citizenship is an instrument of classification: it is a status that divides persons into two groups: citizens and foreigners. Having citizenship entails legal rights, such as the right to enter your country and to live and work there without restriction, the right to vote, the right to hold political office, among others, as well as duties, such as the obligation to serve in the military. Whereas most persons are citizens of one state only, many are citizens of multiple states (and some are a citizen of none).

Academic research and official statistics distinguish populations according to criteria perceived to mark a substantial difference. We are concerned with distinctions based (a) on immigration, be it by the individual or his/her ancestors, and (b) on stable traits assumed to give reason for distinction, such as culture, religion, physiognomy or other biological qualities, in conjunction with their social and economic correlates. The former would be called immigrants, the latter ethnic minorities. We treat these concepts together because they are linked.

### The Netherlands

One in five of the Dutch population is an immigrant, i.e. he or she is born outside the Netherlands, or at least one of the parents. The official statistical term for immigrants, however, used to be the Dutch word ‘allochtoon’ , and for the native population ‘autochtoon’. These terms were introduced in 1989 by the Scientific Council for Governmental Policies (WRR). ‘Allochtoon’ refers to *non-autochthonous*:

“In this report the Council (WRR) interprets *non-autochthonous* as: not of Dutch descent. *Non-autochthonous residents* are foreigners in the legal sense, ex-foreigners who have acquired the Dutch nationality, Dutch who come from former colonies, and their descendants until the third generation, as long as they see themselves as such. An *ethnic minority* is defined as a group of ‘allochtonen’ in a disadvantaged (socio-economic) position” (WRR, 1989, p. 14).

Defining ethnicity and classifying persons on the grounds of ethnic status is, however, not easy. In addition, various “ethnic” indicators can be used for measuring the concept of ethnicity, such as race, country of birth, and nationality (Simon, 2007; Feskens, 2009). In this article we follow the WRR-definition, in which ethnic minorities refer to some disadvantaged groups within the Netherlands. We will focus only on immigrants and their children, i.e. the first and second generation, as we can define this population by a clear and measurable variable: country of birth. The terminology used in publications on immigrants is, however, not always clear and consistent (Nicolaas & Sprangers, 2012). Many terms are used, such as ‘asylum seekers’ , ‘migrants’ , ‘foreigners’ , ‘refugees’ , ‘immigrants’ , ‘status holders’ , and ‘guest labourers’. Often, these terms are used interchangeably. History reveals that some terms are more popular in specific time periods. In the Netherlands, the policy on ethnic minorities dates back to the 1980s. Before the 1980s, the general view was that ‘*guest labourers*’, as they were called, would return to their country of origin and therefore a specific integration policy was not necessary (WRR, 1989). In the Netherlands, the Immigration and Naturalisation Service (IND) is responsible for the implementation of the immigration policy. The term ‘*foreigner*’ is a legal term for a non-Dutch person: someone who does not have the Dutch nationality. Statistics Netherlands (CBS) uses the term ‘*non-Dutch*’ rather than ‘foreigner’. Prior to 1999 CBS used various definitions and terms for immigrants and ethnic minorities. In 1999, CBS in consultation with the Ministry of the Interior, drafted the new definition: a person of whom at least one parent is born abroad. In addition, a further distinction was made between first-generation immigrants (people who were born abroad) and second-generation immigrants (people born in the Netherlands). Another common distinction is between non-western and western immigrants. Non-western immigrants are immigrants whose ethnic background is in one of the countries in Africa, Latin America and Asia (excluding Indonesia and Japan) or Turkey. Western immigrants are immigrants with an ethnic background in Europe (excluding Turkey), North America, Oceania, Indonesia and Japan. For a first-generation immigrant the country is defined as the place where the immigrants were born. To determine the country of a second generation immigrant, the mother’s homeland is used, and in case the mother was born in the Netherlands, the country of origin is defined by the father.

The terms ‘*allochtoon*’ , and for the native population ‘*autochtoon*’ have been under criticism as they offend the immigrants and portray them as citizens not belonging to the Netherlands. Recently, CBS, in close consultation with the WRR (Bovens, Bokhorst, Jennissen, & Engbersen, 2016), decided also to refrain from these terms and use the expressions ‘with a [country/immigrant] background’ , e.g. people with a Turkish background, a Surinamese background, a Somalian background, and also ‘Dutch background’ , indicating the ‘autochtoon’ in their reports (Ooijevaar & Bloemendal, 2016). This makes the Dutch term exactly comparable to the term that has been used in Germany since 2004.

In addition, ‘refugees’ , ‘asylum seekers’ , ‘status holders’ and ‘people with a citizenship’ are often used concepts. A *refugee* is a person who has entered the Netherlands and of whom, based on the 1951 Geneva Convention, it has been established that he or she has a well-founded fear of persecution in the country of origin because of a religious or political conviction, nationality, race or membership of a particular social group. An *asylum seeker* is a person who has entered the Netherlands, has applied for refugee status and is awaiting the decision of Immigration and Naturalisation Service (IND) (Nicolaas & Sprangers, 2012). Asylum seekers can sign up for an asylum application in assigned registration centres, such as Ter Apel and Schiphol. The Central Agency for the Reception of Asylum Seekers (COA) is responsible for the registration of asylum seekers in the refugee centres.^3^ If they are recognized as refugees, they will get a refugee status. Asylum seekers who do not qualify for the refugee status may nevertheless qualify for a residence permit. This may happen, for example, if there are serious grounds for believing that rejected asylum seekers, would face a risk upon returning to their country of origin. *Status holders* are formally recognised asylum seekers whose request has been granted and who received a (temporary) residence permit. *Citizenship*, acquired by birth or naturalization, is a legal bond between a person and the State. Immigrants who wish to naturalize, and become a person with a *citizenship*, should pass the language and integration requirement by successfully completing a formalized naturalization test (introduced in 2003), and should meet other conditions, such as not having a criminal record. In principle, dual citizenship is not allowed in the Netherlands, although there are many exceptions to the renunciation requirement, including being the registered partner of a Dutch national, or when renunciation of the original nationality is not legally possible or cannot be reasonably demanded.

Many of the abovementioned terms to indicate ‘immigrants’ are included in academic and government publications. In the 1980s, publications focussed on policy goals regarding ‘minorities’ showed that there was a lack of reliable data on migrant groups (WRR, 1989). Nowadays, more information is collected and recorded in the Netherlands about the socio-economic position and socio-cultural integration of minorities, by Statistics Netherlands and the Netherlands Institute for Social Research in particular (e.g. Ooijevaar & Bloemendal, 2016; Huijnk, Dagevos, Gijsberts, & Andriessen, 2015).

### Germany

In Germany, surveys often hook onto one particular official concept, viz. *migration background* that has developed into one of the most important categories in state statistics. Until the year 2000, German vs. foreigner was the dominant distinction in public debate and official statistics. While this was formally correct when considered in terms of citizenship, *foreigner* was contested because of its derogatory overtone in colloquial language. Furthermore, the administrative inefficiency of using citizenship became obvious (Verband deutscher Städtestatistiker, 2013): (a) After changes to citizenship law including the introduction of an entitlement to naturalization and a shift from *ius sanguinis* to *ius soli*, naturalization figures rose significantly while there was a perception that the new German citizens were still different and should be distinguished. (b) Aussiedler immigration peaked after 1989. Aussiedler encountered problems similar to other immigrants but were conferred German citizenship upon arrival and thus escaped observation along citizenship lines, and official statistics lacked any means to monitor their fate.

The Federal Office of Statistics then introduced *migration background* in 2004 to unite in one term (a) foreigners and naturalized persons, (b) persons born abroad and immigrated after 1950 regardless of citizenship (thus including Aussiedler), and (c) persons directly born to the above mentioned groups (Statistisches Bundesamt, 2012, p. 6). Subcategories were created based on citizenship, personal migration experience and parent status, but the term is usually treated as a unitary concept. It may also be linked to particular states of origin but this is not equivalent to ethnic categories. Though origins e.g. in Turkey may be singled out, no information on subgroups like Kurds or Yazidi exists. Migration background is widely accepted exactly because it avoids the pitfalls of ethnicity. Further, unlike foreigner, it opens a perspective on monitoring integration beyond the point where citizenship is conferred. It is now the most important concept in political and scientific communication over immigration and integration. Paradoxically, though considered more appropriate by policy makers, it has not generally replaced *foreigner* in state statistics. The law restricts official data collection on migration background to the microcensus, an annual 1% sample of all private households. It should also be noted though that what was originally a purely statistical category has slowly drifted towards reification in public discourse and is increasingly used with as derogatory a connotation as *foreigner* had before (Elrick & Schwartzman, 2015).

*Minority* as an official term has a special meaning in Germany. It applies neither to collectivities resulted from recent immigration nor to disadvantaged groups. It comprises linguistically defined autochthonous groups, viz. Danes, Frisians, Sorbs, and autochthonous Sinti and Roma. The government depicts them in a folkloristic manner with brochures showing traditional costumes and dances (e.g. Bundesministerium des Innern, 2011). Minorities enjoy certain collective rights. They may use their own language in their settlement areas. Their political parties may be exempt from cutoff-points in elections. With the abuse of Sinti lists by Nazi Germany’s police in mind, the state shuns individual registration of minorities. When demanded, e.g. before the Sorb Council elections, resulting data are not accessible for research and so cannot be merged with other registers.

*Asylum seekers* and *refugees* are prominent subgroups of immigrants. The two are not identical. The right to protection for victims of prosecution was codified in the constitutions of both German states after the ordeal of World War II refugees and prior to the 1951 Convention Relating to the Status of Refugees (Geneva Convention). Unlike the latter, these laws restrict asylum to victims of state organized prosecution. Refugees as defined by the Geneva Convention long time enjoyed less rights than asylum holders according to the constitution (Hauser, 2011). Yet, the so-called Qualification Directives 2004/83/EC and 2011/95/EU to harmonize refugee law in the European Community have levelled out these differences.

The asylum procedure results in the categories of recognized vs. refused asylum seekers. The latter category together with visa overstayers and undocumented immigrants who cross borders without official registration of any kind are deemed *illegal residents* (illegal aufhältige Personen) in official language (Sinn, Kreienbrink, & von Loeffelholz, 2005, p. 14). Though known in Germany, the term *sans papiers* (*paperless*) is used less often than in other countries as the public is rather concerned with those who face deportation after orderly registration in an asylum procedure that led to rejection.

*Migration background* is indefinitely attributed to foreign nationals, including grandchildren and great-grandchildren of immigrants proper, who lack German citizenship. Though politically interesting, this category is losing statistical relevance after citizenship law reforms depleted the figures of German-born foreigners. But while children of at least one naturalized parent are associated with *migration background*, grandchildren of at least one naturalized grandparent are no more. The latter category constitutes by far the largest of those with at least one immigrated grandparent.^4^ It is important to note that, as *minority* is inapplicable to immigrant groups, both the political discourse and official statistics lack an agreed-on term for what would elsewhere be called an ethnic minority or the third, fourth etc. generations. Consequently, the major share of the offspring of immigrated families are no longer identified by *migration background* and its technical approximations in the future, and potential particularities they bear thus remain unnoticed. For observers from Anglo-Saxon countries used to ethnic, biologistic, even racial, labels to denote ethnic or visible minorities, this certainly constitutes a gap. While ethnic statistics in general are contentious in continental Europe (Simon, 2007), the main explanation here lies in 20th century history. After the excesses of the Nazi race ideology German society denounces any reference to colour as racial language, and biologistic categories are inconceivable in statistics.

This overview has shown that on the conceptual level the two countries show many similarities. The term ‘with a [country immigrant] background’ was introduced in Germany in 2004, and has recently been adopted in the Netherlands. In the next section the implementation of the concepts in sampling frames will be addressed.

## Sampling

### Population database

There are various ways to implement samples of the groups defined by the categories presented in the previous section. We focus on techniques to draw probability samples that warrant statistical generalisation to target populations. To conduct a survey, a population and a sample are needed. This requires a database with records of various population characteristics, such as age and gender in a specific country. To produce such a database, ideally a population census is required, in which basic characteristics of all individuals are collected for a specific point in time. Countries connected to the United Nations Economic Commission for Europe (UNECE) conduct a census every 10 years on average. To this end, most UNECE countries use personal face-to-face interviews with paper questionnaires. Denmark, Finland, Norway, Sweden, the Netherlands, Austria, and Slovenia build the census on existing registers, sometimes in combination with large-scale surveys, and do not collect additional data (Schulte Nordholt, 2014). Germany is not a ‘register based country’ and has to conduct a census. However, for the last census in 2011 some registers could be used and additional data-collection with surveys were not needed for two thirds of the population.^5^ Germany is no longer a country with a traditional census, but combines registers with additional data-collection. But no matter how the census is implemented in the two countries, one crucial difference exists in that Statistics Netherlands may merge census and population register data while neither authorities nor researchers may do so in Germany. More importantly, although population data are available on the local level, Germany lacks a unitary data infrastructure for sampling immigrants and other national populations.

In the Netherlands, the register-based approach is used not only for financial considerations (interviewing people is much more expensive than using stored data about them), but also because an increasing part of populations is not willing to participate in social surveys. Moreover, in most register countries a database is available containing many variables to draw samples among specific groups in society, including stratified and multi-stage samples.

We will start with an overview of the Netherlands addressing the sampling frames and the possibilities to draw samples on specific immigrant groups. Next, it will be demonstrated that Germany has to cope with many limitations and as such has to search for alternative ways for sampling immigrants. Such alternative sampling methods, are – among others – random digit dialling (telephone numbers), random routes or random walk, time location sampling, capture-recapture, GPS based sampling (within a specific place), and respondent driven sampling or snowball sampling (based on networks) (for a detailed overview see Reichel & Morales, 2017).

### The register approach in the Netherlands

In order to be counted as an immigrant in the Netherlands, a foreign national should be registered in the Municipal Personal Records Database (BRP). In the Netherlands, CBS is the only authority authorised to draw samples based on the BRP and to link registers and surveys into the System of Social Statistical Datasets (Bakker, Van Rooijen, & Van Toor, 2014). As a consequence, almost all national large-scale surveys on immigrants are based on sample designs developed by CBS. An exception is a survey within one single municipality as each municipality has the rights to draw samples from its own BRP.

As a rule, someone is registered in the BRP if he or she “reasonably expects to stay in the Netherlands for half a year, for at least two-thirds of the time” (BRP Law, Article 26). In practice, this is interpreted that a person intends to stay in the Netherlands for the next 4 months. Registration of ‘*asylum seekers*’ and ‘*status holders*’ in the BRP is organized differently than the registration of other immigrants (Nicolaas & Sprangers, 2012). Asylum seekers are usually registered in the BRP – and considered as asylum migrants – when they get a residence permit and move to an assigned dwelling provided by the municipal authorities. According to Article 55 in the BRP, they are not allowed to register, unless they received a residence permit from the IND (Prins & Kuijper, 2007). Asylum seekers who stay in an Asylum Seeker Centre (AZC) will in principle be registered in the BRP after they have stayed for 6 months in the Netherlands, even if they have no residence permit and are still awaiting the granting of their asylum claim. Children who are born in a refugee shelter will be registered in the BRP, even if the parents do not yet have a residence permit and are not registered in the BRP.

CBS links other register and survey data to the BRP. For example, as the migration pattern of non-Dutch immigrants and their motives are not recorded in the BRP, such information is provided to CBS by the IND and linked to the BRP. However, CBS receives only information about the motives of immigrants with a non-Dutch nationality. In addition, the reasons for migration is unknown for most western immigrants. Since May 2006, EU citizens, except for Bulgarians and Romanians in the period 2006–2015, do not need a residence permit if they want to stay in the Netherlands for less or more than 3 months.^6^ This also applies to persons from the EFTA countries (Iceland, Liechtenstein, Norway and Switzerland). Both groups (EU and EFTA countries) still have to officially register and should provide the purpose of their stay in the Netherlands. However, although registration is required for these immigrants, if they do not register it will not affect their right of residence in the Netherlands. Consequently, large groups of immigrants from these countries do not register and do not report to the IND about their motives. No motive was available for about a quarter of EU immigrants to the Netherlands in the period 1995–2005. This share increased in 2006 to almost half to more than 80% in 2009.^7^ If persons move abroad, they are removed from the BRP when the expected length of their stay abroad is at least 8 months. In practise a person will be removed if the expected duration outside the Netherlands is more than two-thirds of the next 12 months (BRP Law, Article 68). However, not everyone reports his or her emigration.

We will elaborate on this issue with a few examples on sampling immigrants, based on stratified samples. A two-stage stratified sampling strategy for the social surveys sample is common practice for the social surveys at CBS. The samples are drawn from subpopulations such as specific immigrant groups, optimizing the comparison of such groups. In the first stage of a two-stage stratified sample, the primary units are selected with probabilities proportional to their size. In a second stage, within the selected primary units, a fixed number of secondary units are selected. For example, in the first stage a random selection out of the municipalities, ensuring that the probability to be included is proportional to the number of inhabitants in the municipalities. In the second stage a random selection of a fixed number of people will be selected within the selected municipalities. A typical example is the selection of 200 out of 430 municipalities in the Netherlands, followed by a selection of 20 people, resulting in a sample of 4000 people.

This strategy is also applied for specific populations such as immigrants. The size of the municipality is determined on the basis of the numbers of ethnic minorities, so that municipalities with a small number of immigrants will have a rather small chance to be drawn. A problem is that the immigrant population, especially when it is based on specific countries of origin, is very unevenly distributed across municipalities in the Netherlands. Municipalities with very few potential sampling units are therefore merged with other municipalities to get adequate numbers for the various immigrant groups. In the Research on Family Formation Young Immigrants (OGJA), the sample was drawn from Turkish and Moroccan youth, aged 18 to 27 years, who were born in the Netherlands (second generation). Starting from a response rate of 40% and a minimum of 900 respondents, a sample size of *n* = 2250 was chosen. The sampling of the first stage revealed that in some small municipalities the required number of 15 people from this target group was not reached. Therefore, some of the municipalities were merged (De Ree & Van Berkel, 2005).

The samples are drawn from the Netherlands Municipal Basic Register which includes basic information of all citizens in the Netherlands. This register serves also as a tool for the production and dissemination of various, e.g. demographic, statistics. Also to this end, the Municipal Basic Register is linked with other registers, e.g. income from the tax authorities, and (large-scale) surveys, such as the Labor Force Surveys. All these data are included in the System of Social Statistical Datasets (SSD), which enables Statistics Netherlands to produce social statistics, and to draw samples based on the whole Dutch population and (very specific) subpopulations. Such a subpopulation could for example be all people with the following characteristics: woman, aged 18–24, living in rural areas, and non-western immigrant.^8^

For such tailored-made sampling strategies, also other registers and large-scale surveys are used apart from the municipality registers. In addition to BRP, such a useful register is the WNB (in Dutch: ‘werknemersbestand’), that encompasses data on employee tax payments from all immigrants that are legally employed in the Netherlands. Moreover, the Registration for Non-residents in the Netherlands (RNI) includes all persons who come to the Netherlands to study or work for less than 4 months and who need a Citizen Service Number (BSN), and the Dutch police authorities make use of 27 Recognize Service System registers (HKS) with information on crime suspects. Also some estimates on the illegal immigration are available, based on other registrations such as HKS. Using capture-recapture techniques, various studies show that a substantial number of immigrants from Eastern Europe are not registered (Van der Heijden, Cruyff, & Van Gils, 2013; Bakker, Gerritse, & Van der Heijden, 2015).^9^^,^ 10 Other figures, in which WNB and BRP are linked, reveal that only one in four of the employees from Central and Eastern European countries are registered in the BRP (CBS, 2011).

CBS also works together with other institutes, the Netherlands Institute for Social Research (SCP) in particular. Within the framework of the Research on Processes of Social and Cultural Integration (SCIP), a sample was drawn of 1397 first generation recently immigrated Romanian migrants aged 18–65, registered in the municipalities between January, 1, 2013 and October, 1, 2014. The sample was based on municipalities in the Municipal Basic Register in which at least 25 migrants were registered in that specific period; this concerned in total 26 out of 405 municipalities.

An advance letter was sent in the Romanian language, in which an incentive of 10 euro was promised for a completed face-to-face interview (Gijsberts & Lubbers, 2015a).^11^ Out of the 1397 selected Romanian migrants 356 were interviewed. This resulted in a response rate of 25%. The main reasons for non-response were ‘nobody at-home’ (12%), ‘moved abroad’ (19%), and ‘does not live on the provided address’ (30%). The refusal rate was 9%. If we would leave out the framework errors, the response rate would increase substantially to 51%. Both sampling and non-response are two crucial aspects of the Total Survey Error, which also includes the collection, processing and analysis of the data (Biemer, 2010) This begs the question how much bias will be introduced due to the non-response based on the framework errors and other reasons. We may expect that the bias is substantial as respondents will differ from non-respondents. The bias is, however, difficult to assess as information on the target variables based on all Romanian migrants is lacking. If a key characteristic in a register would be available that is strongly related to the target variables in the survey, the estimates from the survey could be compared with the register information and included in weighing models in order to adjust for the non-response bias. Such an example would be voter turnout enabling a rather strong adjustment in many target characteristics in the Dutch Parliamentary Election Study (Schmeets, 2015).

The above example on the Romanian immigrants clearly demonstrates that many recent immigrants and ‘undocumented migrants’ or ‘paperless’ are not registered, and hence they are not included in the sample. In addition, the sample may contain many immigrants that have moved abroad or to another municipality, but are still included in the (wrong) municipal register. A further drawback is that the sample is based on 26 municipalities only, and it remains unclear whether these municipalities are a good reflection of all Romanian immigrants, including those living in the other 378 municipalities existing then.^12^ For cost-efficiency reasons a tailored research on specific ethnic minority groups will exclude some municipalities from the sample, simply because it is too expensive to get in contact with a very few people. Another strategy would be to select the Romanian immigrants from large-scale surveys. However, the Romanian population in the Netherlands is 0.11%, which will bring us some 80 Romanian respondents in the Labour Force Survey, assuming an average response rate (see also Huddleston et al., 2013, pp. 33–34). Merging data will only be an option if we will compile data from 2014 onwards (before the proportion was even lower). To achieve the same number compared to the SCIP-sample, it will take at least some 5 years. The randomized probability samples based on population registers is probably the best sampling design to draw generalised conclusions. Non-probability samples, such as quota samples, snowball samples and convenience samples are not an option for CBS. Other techniques based on probability samples, such as network samples, and random digit dialling are also not used as the population registers are available and have many merits in comparison to other techniques. It also serves as the backbone for the System of Social Statistical Datasets, and tailored weights can be produced to reduce the non-response bias. However, as shown, sampling of immigrants is not easy and results in low response rates, the newcomers in particular. Such hard-to-survey groups call for another approach in which also other registers than the BRP will be used to build a sampling frame. If we aim to include all immigrants who stay in the Netherlands temporarily and for a rather short period, such as seasonal workers in the agricultural economy, we may use the RNI and WNB. Furthermore, data from the ‘Asylum Seekers Centres’ are needed to cover the asylum seekers and refugees.

### The population register and its alternatives in Germany

The sampling strategies conducted by CBS cannot be implemented in Germany. Sampling in Germany has to cope with three constraints: (1) the absence of a national register; (2) the regional structure; (3) the legal system;.

1. Germany has no national population register and thus no complete unitary sampling frame. Each community (district or town) maintains its own register. Hence sampling imposes compromises at the level of the sampling frame.
2. In Germany, all major immigrant populations settle in a multitude of regions and are less segregated within cities than in other countries (Schönwälder & Söhn, 2009). This has to do with the regionally scattered economic structures attracting labour migrants and the legally stipulated regional dispersion of Aussiedler and refugees. All sampling techniques face a trade-off between extended geographic scope for adequate socio-structural diversity and cost-efficiency.
3. The definition by the Federal Statistical Office cannot adequately be implemented outside the microcensus due to lack of data which on its part has legal reasons. German data protection law restricts data collection and use to legally stipulated purposes. Authorities must not collect data unless state functions hinge on them, and mere statistics are no sufficient purpose. *Migration background* builds on variables not otherwise necessary for public administration responsibilities. It also depends on data pertaining to parents, but links to third persons fall under even more severe restrictions. An explicit legal framework had to be created for the incorporation of *migration background* into microcensuses. Unfortunately, it covers no other databases. Thus notably the population register, which we shall discuss below as the best possible sampling frame, lacks crucial information. Merging census and population data is forbidden, even to authorities. The definition can therefore only be approximated with non-microcensus data (Verband Deutscher Städtestatistiker, 2013) and citizenship continues to serve as an important identifier.

In Germany, the population register constitutes the most comprehensive sampling frame. Each local authority population register contains almost the entire population living within its territory, regardless of citizenship and right of residence. Registration is compulsory no later than 2 weeks after moving in, and fines can be imposed for infringement. The register excludes members of foreign armed forces, foreign diplomats, and some categories of paperless migrants. Furthermore, people who move within Germany without registering create a mismatch between registered and resident population, and people who move abroad without deregistering, e.g. pensioned migrant workers, lead to a net over-counting. Without registration, access to social services is impossible and finding legal jobs is more difficult. Thus despite many discrepancies, the population register is the best available representation of both the overall population and the population with migration background; it can be said to exclude only a small part of the immigrant population.

The use of population register data is governed by the Registration Act which includes an interface for data disclosure to outsiders. Universities may be supplied with name, address, date and place of birth, and current citizenship. With certain restrictions, this permits to infer migration background. It is easy to identify non-citizens, but not naturalised citizens unless they hold dual citizenship. Most Germans with at least one other citizenship fulfil the criteria of migration background and can be identified directly. First-generation immigrants can be identified on the basis of place of birth. The register contains no information on the age and date of arrival in Germany. Generation status must be requested directly in surveys. Mixed-nationality marriages cannot usually be identified on the basis of different citizenship within a family. Information about family relationships between spouses and other adults within the same household is contained but cannot be disclosed. For minor children, information including nationality can be obtained on the legal guardians, usually meaning the parents. No information about residence permits, asylum status or migration motives is available. Sampling refugees therefore cannot be implemented directly in the population register.

Register data are dispersed over several thousand municipal authorities. Sampling strategies therefore either take a two stage approach with a sample of communities followed by a sample of individuals, or they use alternative techniques, either with or without selection frames. Typical two stage samples to achieve representativeness would include over 100 local level sampling points. Their procurement is complex and time-consuming (Albers, 1997). The permissibility of data release must be negotiated with each individual local authority. Charging programming costs for complex selections is at the deliberation of the authorities. The consequence is that geographically extensive sampling can currently only be conducted with an extraordinary expenditure of resources.

The population register neither provides all desirable identifiers discussed in chapter 3 nor does it map the German *migration background* concept directly but it comprises core information.^13^ Drawing on the essential characteristic of citizenship is technically uncomplicated. For Aussiedler, who are usually German citizens, it is unsuitable, however. A considerable proportion, including the second generation, are identifiable through dual citizenship of their country of origin (Salentin, 2007). German citizens make up 54.9% of the population with migration background (Statistisches Bundesamt, 2012, p. 56ff.). For most other immigrated groups, the proportion of German citizens is smaller, with wide variations; citizens of EU member-states are less likely to apply for citizenship, refugees more likely (Woellert, Kröhnert, Sippel, & Klingholz, 2009).

Restricting the scope to non-citizens creates a qualitative distortion of the social structure of the sample. Naturalized citizens exhibit better socioeconomic parameters and more strongly assimilated attitudes than noncitizens from the same region of origin. Noncitizen samples systematically exclude the more successful immigrants (Weinmann, Becher, & Babka von Gostomski, 2012, p. 6). Naturalization is used as a dependent and independent variable of integration research that must not be allowed to affect the sampling.

The place or country of birth is a reliable indicator of immigration. It perfectly identifies first generation Aussiedler and other migrants who possess only German citizenship. That makes it a crucial variable in the population register. But Aussiedler descendants can no longer be identified unless parental data can be accessed. Birthplace also is an uncoded string variable in the population register. Utilization requires country-specific directories of place names and programming effort (Salentin, 2007).

In Germany like in most countries the personal names (first and family names) of immigrants differ from those of the native population. Names may be used for drawing samples. Indeed, a large body of German research is based on samples selected namewise from the population register or from phone books. Some fundamental problems are known: Name assimilation takes place and intermarriages blur the demarcation of name boundaries (Mateos, 2007). Compiling comprehensive name directories is almost impossible due to their sheer size. For countries of origin with similar name distributions name-based selection methods are assumed to fail (Humpert & Schneiderheinze, 2000, p. 40; Groenewold & Lessard-Phillips, 2012, p. 42).

Among the clear drawbacks of the population register is the lack of information on legal and refugee status, year of immigration and age at immigration (and thus generation), former citizenships, occupation, religion and self-declared ethnicity.

Taken all the limitations of the population register in terms of access and regional scope into consideration, Germany has to search for other options to draw samples on immigrants. In total four strategies will be explored, all having specific drawbacks.

The first option would be the *central Register of Foreigners*. The Central Register of Foreigners accumulates data on all foreigners living in Germany. It includes all refugee data. At first sight it might serve as a nationwide sampling frame. But there is no legal basis for using its data in academic research. Vogel and Aßner (2011) who were entitled to use the register for an official survey report a cumulative overrecording. When a person is naturalised their data are immediately deleted; and as explained earlier, naturalised citizens and non-citizens differ structurally. The Central Register of Foreigners is therefore no suitable sampling frame.

A second option is the *telephone directory*. Machine-readable telephone directories are available online and on CD. They have frequently been used for selection using name-based methods. They yield household samples and require a subsequent random selection of a target person. The attractions of this approach are ease of access at very low cost and national coverage in a homogeneous data set. The existence of the telephone number facilitates telephone surveying. But (a) it is unclear if using participant data for surveys is permissible because under German law personal data may not be processed without consent. Phone owners consent to publication of their data for telecommunication purposes only. (b) Households that have a landline but no telephone directory entry cause distortion (Deutschmann & Häder, 2002; Häder, 1996): unlisted subscribers are disproportionately low-income households, live in large cities, are more mobile, younger, and tenants rather than owner-occupiers. As for the telephone directory entries of immigrants, further misrepresentation was observed. Sauer and Goldberg (2001, p. 29) found a surplus of middle age groups, singles, self-employed, and large households in the telephone directory vis-à-vis the microcensus. Comparing telephone directory samples of several nationalities with the microcensus, Santacreu Fernández, Rother, and Braun (2006) found discrepancies in the distribution of gender, marital status, age, age at migration, migration period, education, and employment status. Salentin (2002) attempted to re-identify a population register sample in the telephone directory. This was possible for 65% of the people of Turkish origin but only 40% of those from Serbia. (c) The telephone directory is shrinking rapidly. The total number of entries fell from 40 millions in 1998 to 22.9 millions in 2015, and the number identified as being of Turkish origin decreased by 54.4% (Wittlif & Beigang, 2016, p. 9). According to Salentin (2014, p. 36) just 36% of households were listed in 2011/2012. (d) Apart from the names, telephone directory entries contain no indicators of migration background. The scope of the resulting samples is thus limited. These are certainly strong arguments against using the telephone directory beyond merely exploratory studies.

A third possibility is *area (random route) sampling*. Before 2000, when not every household possessed a telephone, in particular in the eastern parts of Germany, a good approximation of a random sample of the population was achieved by contacting subjects directly in their homes guided by routing instructions. This is still so, and it works for the population with migration background. Given a proportion of 22.5% of all inhabitants,^14^ that group is well represented in the resulting samples, without any special measures, and with a manageable screening effort, which is inverse to the proportion of the population. But as soon as country-specific groups are to be identified, such samples become highly inefficient. For example, for every person of Italian origin (population in Germany 780,000), 128 contacts would be required. Concentrating fieldwork in areas with higher proportions of the target group is less efficient in Germany than in other countries because immigrants here are comparatively unsegregated (Schönwälder & Söhn, 2009). Without large budgets, immigrant samples using random walk will result in poor sample quality.

*Phone number dialling is the fourth possibility*. Sampling by controlled random dialling of landline numbers (Gabler & Häder, 1997) is, however, similarly deficient. Increasingly, Germans abandon landline telephones. Among them single-person households, men, under-30s, low-income groups, and people living in eastern Germany and Berlin are overrepresented (Mohorko, De Leeuw, & Hox, 2013, Tables A1, B1; European Commission, 2010, p. 52; Gabler & Häder, 1997). The screening effort for smaller populations is considerable, quite apart from identification problems. The issues are similar for dialling cell-phone numbers, although this compensates the growing coverage bias of the landline network, and for dual frame approaches (Callegaro, Ayhan, Gabler, Haeder, & Villar, 2011). For those reasons these methods will not be discussed further. Recently a promising novel approach was tested. Customers of phone service providers with reduced rates for foreign calls into particular countries were identified by number prefixes and screened for migration background (Wittlif & Beigang, 2016, p. 9f) which yielded a high proportion of foreign born persons. While this approach alone may introduce an intolerable bias, it could be combined with a name based selection from the phone directory and random dialling into a cost-efficient alternative to population register sampling.

For special migrant populations defined by religious or ethnic traits, legal status, language, occupation or simply with low counts all techniques create screening expenses beyond reasonable dimensions. To reach these populations researchers may abandon established probability sampling techniques and turn to the options discussed by Reichel and Morales (2017). But though their practicability has been proven in several countries, further research is necessary before they can be recommended.

## Conclusions

The Netherlands and Germany have many basic points pertaining to sampling of immigrants in common though with limitations. Both use the concept of migration background based on the country of birth of an individual or his/her parents. They focus on immigrants merging into resident populations in the third generation and do not envisage permanent minorities. Both renounce subjective, ethnic and biologistic categories to describe subpopulations. Differences exist in detail, as e.g. Germany includes foreigners irrespective of place of birth of the individual or the ancestors whereas the Netherlands looks at parents’ birth place at most. This makes a quantitative difference, since Germany has low naturalization rates. This begs the question whether the immigrant population captured by Dutch concepts is more integrated than those comprised by the German concept. For both countries, is has been demonstrated that naturalisation matters in regard to paid jobs, in particular for immigrants stemming from less developed countries (Peters, Vink, & Schmeets, 2017; Steinhardt, 2012). To become a Dutch citizen, a naturalisation test have to be passed in which their Dutch language skills (A2 level) is tested. As a consequence, it is possible that immigrants also participate more often in surveys with single lingual questionnaires in the Netherlands than in Germany.

In principle, the population register can serve as a sampling frame in both countries. We consider a two-stage strategy in which first municipalities and secondly individuals are randomly sampled the best recommendation for the Netherlands and Germany. But the similarity ends when it comes to implementation. Register organization is centralized in the Netherlands and decentralised in Germany. While the selection of municipalities can be effected within a unitary Dutch data frame, German municipalities have to be approached individually. Dutch law allows record linkage between official population, immigrant, tax, and labour force data while German data protection rules this out. This results in a powerful and comfortable sampling frame in the Netherlands and a myriad of tiny local-level frames in Germany. The Dutch System of Social Statistical Datasets provides direct access to types of migrants defined by legal status or migration motive, while sampling in Germany has to get along with such distal identifiers as citizenship and place of birth. On the other hand, while the register excludes only small numbers in Germany, undercoverage in the Netherlands extends to many short term migrants and sizable portions of the citizens of European and other states. Harmonisation of immigrant statistics is thus less a problem at the level of concepts than in implementation. The differences are deeply rooted in the organisation of the states and comparable sampling procedures are doomed to remain wishful thinking. This being said, and with the problems described in “Sampling” section in mind, we shall wind up with recommendations for the best possible implementation.

In the Netherlands, as shown, it is hard to sample immigrants due to various reasons. The sample is drawn from the BRP which includes only the registered immigrants. Many immigrants are not included in this register, such as people who intend to stay for less than 4 months. Another example is the former asylum seekers whose claim has been rejected. Immigrants from EU-countries should register after a stay of 3 months. All EU-citizens who intend to stay for more than four out of 6 months should register at the BRP at the municipality of residence. However, a substantial number does not register, as revealed in the survey on Polish migrants (Gijsberts & Lubbers, 2015b). Furthermore, many EU-citizens do not bother asking for a residence permit at the Immigration and Naturalisation Service (IND), since that does not affect their entitlements, unlike what is the case for non-EU citizens. However, if EU-citizens do not register at the IND this will often have no impact on their right of residence. Hence, a substantial number EU-immigrants is not recorded in the database and will not be part of the sample frame. In addition, some people who left the country again might still be included in the database. Statistics Netherlands assesses, with the help of the local authorities, how many people have left the country without unregistering from the BRP. These data are published separately, and added to the number of registered emigrants. There are, however, a substantial number of – often elderly – people with an immigration background who ‘commute’ on semi-annual basis between the Netherlands and their country of origin without unregistering themselves from the BRP. As a consequence, there are many errors in the sampling frame which will result in low response rates, and non-response bias. This calls for an amendment in the definition of the concept “immigrants”, in which some immigrant groups are being excluded as they will not be found in the BPR. Another alley would be to improve the sample frame with information based on other registers (Bakker & Kuijvenhoven, 2010).

On top of the non-response due to sampling frame errors, immigrants are hard-to-survey groups which will reduce the response even further. With tailored strategies for hard-to-survey groups, the cooperation of immigrants in surveys need to be enhanced (Feskens, 2009; Kappelhof, 2015; Luiten, 2013). Improving sample frame and data-collection strategies will be a first step into higher quality of surveys among immigrants in the Netherlands.

There is no comfortable choice for surveying immigrants in Germany either. As long as the target population is simply characterized by migration background without further differentiation, established procedures for the entire population, viz. random route sampling and mixed frame telephone interviews, are sufficient. The target population constitutes 21% of the total population, and screening expenses will often be permissible. When specific groups are targeted, the gold standard consists of a two stage procedure. First, a large sample of cities is selected that covers the diversity of socioeconomic conditions across the country. Second, individuals are drawn from population registers using citizenship, place of birth and name. Though groups like Aussiedler offspring with typical German names fail to be identified, this procedure captures very large parts of the population with migration background. However, expenses and manpower demand are considerable. Many surveys have implemented more convenient and economically practical schemes, though at the expense of sample quality. Limitation to a few large cities where population registers or area sampling are applied leads to cluster effects and a bias towards metropolitan and more segregated living conditions. When foreign citizenship is substituted for migration background, a massive sampling bias lurks. Telephone directory samples with name-based immigrant recognition are the cheapest variant. Their popularity was high in the past but has declined when the selection frame started to shrink. Today it demands for combination with other techniques such as targeted random dialling. All other techniques are either legally inaccessible for academic researchers or no less laborious.

Achieving a harmonised data-collection on immigrants for Germany and the Netherlands will be a major challenge. A harmonized approach across Europe will, however, even probably be a bridge too far.

## Footnotes

1 See, e.g.: http://statline.cbs.nl/Statweb/publication/?DM=SLNL&PA=37713&D1=0-2&D2=0&D3=0-5,8,11,14,18,20-22,24,27,29,31-33,35-36,38-42,49,54&D4=0&D5=0,4,14,18-20&VW=T.

2 press release of the Ministry of the Interior, 11 January 2017.

3 If the application is rejected in the general asylum procedure, asylum seekers have to leave the country within 4 weeks.

4 press release, Federal Office of Statistics, August 1, 2017.

5 See: https://www.zensus2011.de/EN/2011Census/Methodology/Methodology_node.html.

6 After accessing to the EU in 2007, for Bulgarians and Romanians there was a transition period for 7 years. They needed a work permit and there was a ceiling to the number of permits issued. However if they could proof they had an income of their own, they also could become a resident.

7 Using information from the BRP – such as the year of establishment of the partner, the year they were married or lived together, the age at the time of immigration – the number of family migrants (family reunification and family formation) has been estimated for migrants from EU- and EFTA countries. The other, unknown migration patterns (work and study) are imputed.

8 Statistics Netherlands makes use of information from fixed telephone lines and mobile numbers (also retrieved from other companies). However, not all persons have registered telephone numbers, and using Computer Assisted Telephone Interviewing (CATI) gets more and more problematic.

9 See: https://statline.cbs.nl/Statweb/publication/?DM=SLNL&PA=82266ned&D1=0&D2=0&D3=0&D4=0-1,3,6,8,11-16,18-19,23,25,27,31,33,35,l&D5=a&VW=T for an overview in the period 2008–2011. Other information refers to people from other countries who are sent back at the border in the period 2008–2015 (see: http://statline.cbs.nl/Statweb/publication/?DM=SLNL&PA=82268ned&D1=0&D2=0-5,9-11,14-23,26&D3=a&HDR=T,G2&STB=G1&VW=T).

10 Since 2009, using the capture-re-capture method, the homeless population has been estimated (some 31 thousand in 2015).

11 See: https://www.scp.nl/Onderzoek/Bronnen/Beknopte_onderzoeksbeschrijvingen/Socio_Cultural_Integration_Processes_among_new_immigrants_in_Europe_SCIP.

12 Meanwhile, as of January, 1, 2017, the total number of municipalities has been further reduced to 388.

13 For a detailed discussion: Salentin (2014).

14 press release, Federal Office of Statistics, August 1, 2017.
